# Understanding infant stress in neonatal and pediatric intensive care: a scoping review

**DOI:** 10.1007/s44253-025-00083-4

**Published:** 2025-10-21

**Authors:** Christine Riley, Christopher Mastropietro, Ashley Darcy-Mahoney, Christine Pintz, Quiping Pearl Zhou, Pamela S. Hinds

**Affiliations:** 1 Children’s National Hospital (Division of Cardiac Critical Care), George Washington University – School of Medicine and Health Sciences, Washington, DC USA; 2 Riley Hospital for Children at Indiana University Health, Indiana University School of Medicine (Department of Pediatrics, Division of Critical Care), Indianapolis, IN USA; 3 George Washington University – School of Nursing, Washington, DC USA; 4 Children’s National Hospital (Department of Nursing Research), George Washington University – School of Medicine and Health Sciences, Washington, DC United States

**Keywords:** Infant stressors, Environmental stressors, Care environment, Pediatric intensive care, Neonatal intensive care

## Abstract

Infants in intensive care environments encounter numerous stressors which may overlap or occur in close proximity. Recent literature suggests stressful encounters in the neonatal or early infant period are harmful to physiological, functional, and structural systems, both acutely and longitudinally. Identifying these stressors and assessing the stress burden in this vulnerable population is crucial for developing care models that minimize unnecessary stress, thereby enhancing recovery and survival. This scoping review identified sources of stress encountered by infants hospitalized in intensive care environments, as reported in empiric literature. A total of 51 studies met eligibility criteria. Identified stressors were categorized into environmental stressors, stressors associated with routine care, and stressors associated with noxious or painful procedures. Studies predominately focused on premature infants in neonatal intensive care units; research on stressors in general pediatric intensive care units or among infants with congenital heart disease or other congenital anomalies is lacking. Evaluation of infant stress varied across studies, though most utilized vital sign alterations, biochemical markers, biophysical assessment, or observational scales by clinician report. Across studies, findings suggest stress experienced by infants in intensive care settings may contribute to physiological disruptions and developmental vulnerabilities.

## Introduction

Infants hospitalized in intensives care environments are subjected to numerous repeated stressful stimuli posing significant risks to physiological, functional, and structural systems. Increasingly, researchers are recognizing that an infant’s intense reaction to stress is not only acutely harmful but carries lasting negative impact [1–3]. Stress encountered in this critical period of development may attenuate gene expression, autonomic nervous system regulation and response, hypothalamic–pituitary–adrenal axis function, and immune function [2–5]. Animal models simulating stressors encountered by infants in intensive care settings demonstrate slowed growth, negative impacts on both acute physiological and long-term neurodevelopmental indices, and increased mortality [6]. Similarly in human infants, the impact of these stressors is not limited to the initial physiological instability following stress exposure but rather can create permanent alterations in physiological functioning such as impaired brain maturation and impaired somatic growth [4, 6–8].

Examining sources of stress encountered by infants in intensive care environments is crucial for enhancing their care and promoting better health outcomes. While stress can be defined as the physiological, psychological, and behavioral response to a stimulus or demand, which may also be physical or psychological [9, 10], stressors are defined as any stimulus that disrupts homeostasis that triggers a stress response [10]. The concept of Infant Medical Trauma identifies stress as an overarching factor, specifying bidirectional relationships with but distinct from the experience of pain and parental separation [11]. Identification of stressors encountered by infants in intensive care environments and measurement of stress burden in this vulnerable population are fundamental to creating care models that minimize or remove unnecessary stressors and optimize infant recovery. The aim of this review is to identify and summarize sources of infant stress, beyond disease processes, encountered in by infants in intensive care environments.

## Methods

Scoping review methodology was utilized to locate and review existing evidence. Scoping reviews aim to determine the existing scope or coverage of a specific topic in current literature, provide an estimation of the volume of literature available, and an assessment of overarching findings [12]. In contrast to a systematic review, scoping reviews provide an overview of current evidence rather than aiming to answer a specific practice-related question and are therefore most appropriate when addressing emerging bodies of evidence [12, 13] This is the appropriate methodology to achieve this review’s aim to explore existing literature on sources of stress encountered by hospitalize infants in critical care environments.

### Search strategy

This scoping review of peer-reviewed manuscripts published in or translated to English was completed following a comprehensive search utilizing both medical search headings (MeSH) and title/abstract keywords in PubMed from inception to March 2025. Subsequently, a bibliographic review of pertinent studies identified a complete list of references. Study terms related to stress (e.g., “stress, physiological”, “environmental stress”), infant population (e.g., “infant, newborn”, “infant”, “heart defect, congenital”), and critical care clinical settings (e.g., “neonatal intensive care unit”, “critical care”, “cardiac critical care”) defined the search strategy.

### Inclusion and exclusion criteria

Studies that empirically evaluated stressors experienced by infants in the intensive care environment were included. Empirical evaluation was defined as the collection of observable or measurable markers of infant stress through experimental or observational methods. Studies conducted in animal models, well infants, or in hospitalized infants outside of the intensive care unit (e.g. operating room, emergency department, acute care wards) were excluded as this review focused on stressors encountered by infants in intensive care settings. Studies testing care models aimed at stress reduction (e.g., clustered care), and studies focused on specific stress reduction interventions (e.g., cycled light, music therapy, kangaroo care) were excluded as this review focused on empirically identified stressors rather than stress reduction interventions. Instrumentation studies reporting development of stress scoring tools or development of conceptual models of infant stress without subsequent empirical testing were excluded. Studies of pain, discomfort, or specific clinical conditions without identification of these experiences or conditions as a source of infant stress were excluded.

### Data extraction

Data were systematically extracted from studies meeting inclusion criteria using a standardized template capturing both study characteristics and findings. Measures infant stress and infant stress response were reviewed across studies, as were details of the study sample population. Identified stressors were grouped into broader categories.

## Results

**Fig. 1:**
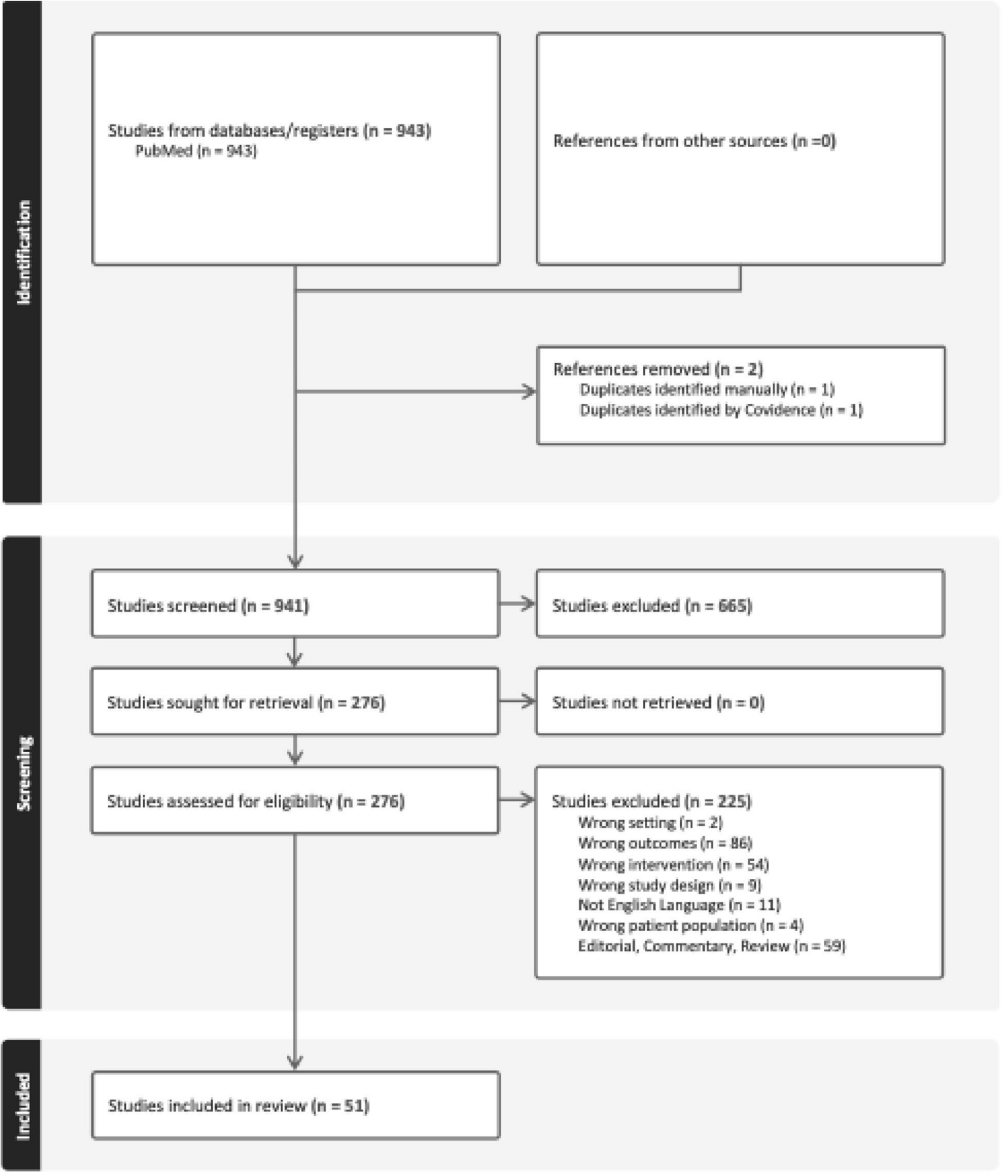
PRISMA flow diagram of structured literature search

**Fig. 2:**
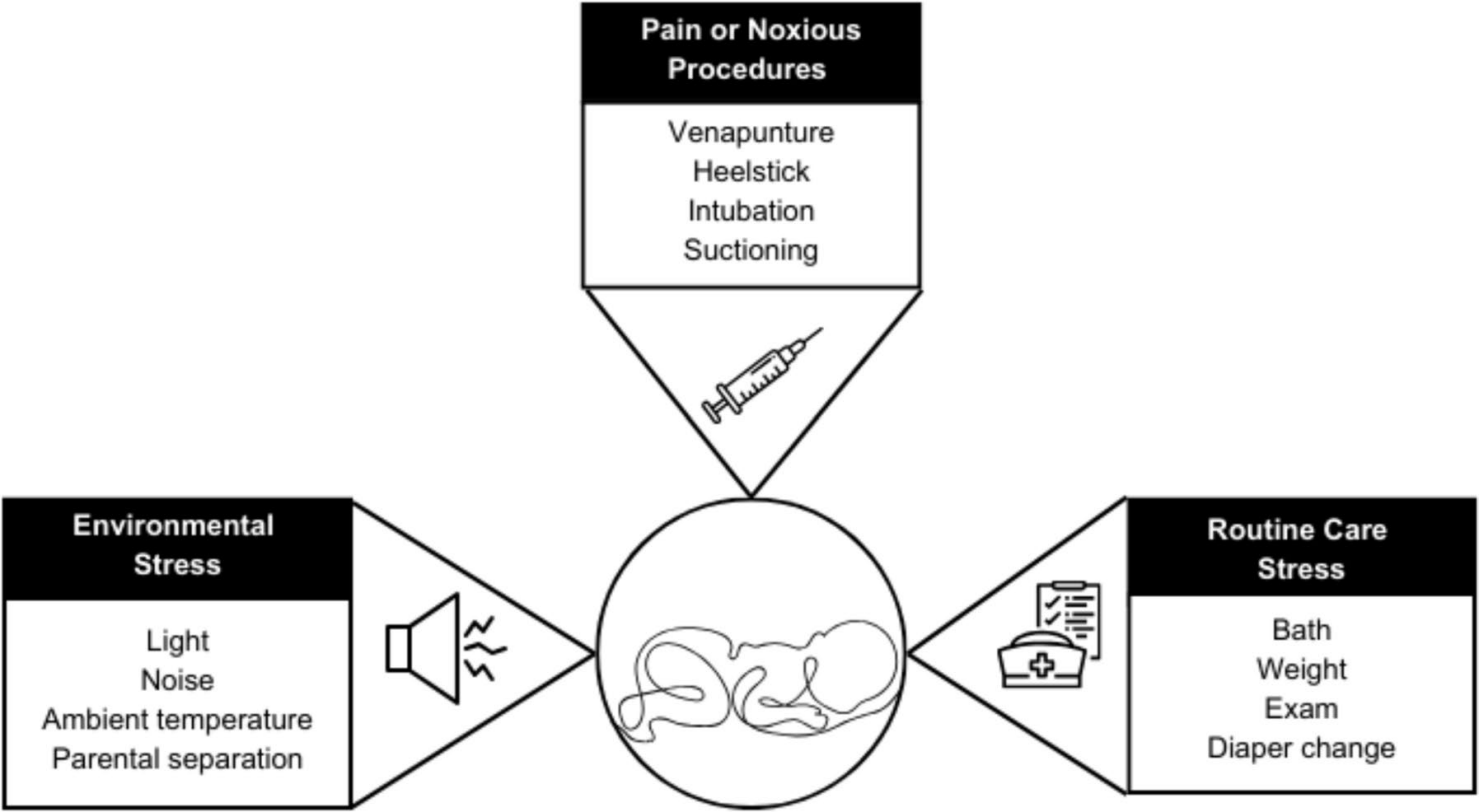
Identified stressors in this review were categorized into environmental, routine care, and noxious procedures. These stressors contribute to the cumulative stress burden of infants cared for in intensive care settings

The search strategy identified 941 articles. Following removal of duplicate citations, 665 articles were screened. Following title and abstract screening, 275 articles were retained which underwent full text stressing. Fifty-one studies remained following full-text assessment for eligibility. Results of study selection are detailed in Fig. 1 (Fig. 1 PRISMA flow diagram of structured literature search). Identified infant stressors in the intensive care clinical care environment were categorized according to the following themes: environmental stressors, stressors associated with routine care, and stressors associated with noxious procedures or pain as shown in Fig. 2. (Fig. 2. Identified stressors in this review were categorized into environmental, routine care, and noxious procedures). A summary of included studies is presented in Table 1. Table 2 presents identified stressors by category.

**Table 1:** Summary of included studies. Findings of scoping review on sources of stress encountered by infants hospitalized in intensive care environments detailing author, year, and brief description of sample and reported findings

*Author (Year)*	Sample description (n)	Key Findings
Zahr (1995) [14]	• Premature infants: weight 480-1930 g, 23 to 37 weeks GA (*n* = 55)	• Infants in open warmer were subjected to four times as many nursing interventions as infants in incubators • Average of 8.5 episodes of handling due to nursing interventions in the 4 h observation period • Nursing interventions and noise resulted in significant behavioral and physiological stress response
Bernert (1997) [15]	• Suctioning periods (*n* = 20) in preterm babies 26–32 weeks GA (*n* = 13)	• Arterial desaturation and cerebral deoxygenation with suctioning • HR decreased immediately after suctioning, but subsequently there was a long-lasting increase in HR • Changes in arterial saturation, deoxygenated hemoglobin, and oxygenated hemoglobin, and increased HR were behavioral-state dependent
Neu (1997) [16]	• Preterm infants (*n* = 13)	• Increased physiologic distress, decreased motor organization, less effective self-regulation with unswaddled weighing
Peters (1998) [17]	• Preterm infants (*n* = 13)	• Physiological and behavioral disruptions during bath and following, including increased HR, decreased in O_2_ saturation, cardiac oxygen demand, behavioral motor cues • 69% of infants required increased concentrations of ambient oxygen following bath
Quinn (1998) [18]	• Preterm infants (*n* = 30)	• Reduced adrenaline concentrations with patient triggered ventilation • No changes in noradrenaline concentrations or clinical distress score • Higher adrenaline and noradrenaline concentrations in non-survivors
Lee (2002) [19]	• Preterm infants: 27–36 weeks GA, between 1000—2500 g (*n* = 40)	• Sponge bathing associated with change in cardiac state (R–R intervals, vagal tone, HR) • No differences on behavioral states
Matsubasa (2002) [20]	• VLBW infant (*n* = 50), normal controls (*n* = 26)	• Infants on mechanical ventilation experienced more intense oxidative stress • No variation in oxidative stress with ambient oxygen concentration
Trapanotto (2004) [21]	• Healthy newborns: 24 and 72 h after birth (*n* = 21)	• Significant and continuous increase in EMG activity in response to noisy stimuli • EMG remained disturbed in recovery phase • Significant differences between types of noise; greatest EMG response to intense noise
Grunau (2005) [22]	• Preterm infants: born ≤ 32 weeks gestation (*n* = 87)	• Greater cumulative exposure to skin breaking procedures associated with lower cortisol response and lower facial reactivity, independent of early illness severity and morphine exposure
Ahn (2006) [23]	• Premature infants (*n* = 54)	• Significant change between mean baseline pain scores and scores during NICU procedures*
Liaw (2006) [24]	• Preterm infants (*n* = 12, 192 observations)	• Stress behaviors increased from baseline during the active phases of the bathing process • Infants found to be overstressed as a bath proceeded and distress decreased post-bath
Mörelius (2006) [25]	• Premature infants: 23–38 weeks GA (n = 39); healthy full-term newborns (*n* = 30)	• Both groups showed an increased pain response to a standardized diaper change • Higher stress scores during diaper change and more sustained increases following • Higher median baseline salivary cortisol levels • Salivary cortisol decreased in response to the second nappy change in NICU infants
Holsti (2007) [26]	• Preterm infants: < 32 weeks GA (*n* = 54)	• All infants demonstrated increase stress response when routine clustered care was preceded by a painful procedure compared to a clustered care preceded by a rest period
Pineles (2007) [27]	• Healthy preterm infants: less than 48 h of mechanical ventilation (*n* = 22)	• HR and behavioral agitation significantly increased during each heel stick compared to baseline • HR response was larger to the third heel stick compared to the first two procedures • Sensitization was observed in infant HR, but not in behavioral responses
Yamada (2007) [28]	• Premature infants: > 25 weeks GA at birth, hospitalized a minimum of 30 days (n = 60)	• Higher hair cortisol levels in hospitalized infants compared to term infants • Total ventilator days associated with hair cortisol levels; 21% variance in cortisol explained by total ventilator days
Carbajal (2008) [29]	• Neonates admitted to tertiary care centers, procedures documented in first 14 days of admission (*n* = 430)	• 141 ± 107 mean stressful and painful procedures per neonate • 16 ± 9 procedures per day of hospitalization • 98 ± 78 painful procedures per neonate • 12 ± 8 painful procedures per day of hospitalization • 79% of the documented painful procedures were not accompanied by analgesia
Lucas-Thompson (2008) [30]	• Healthy premature infants (*n* = 49)	• Both GA groups (28–31 weeks, 32–34 weeks) displayed behavioral and cardiovascular stress response to heel stick • Infants born at younger GA were more physiologically reactive
Padhye (2009) [31]	• VLBW infants admitted < 30 weeks post-menstrual age (*n* = 38), 205 observations	• Decreased HRV in response to blood draw procedure • Venipuncture and mechanical ventilation attenuated the HRV response to pain
Peng (2009) [32]	• Preterm infants: < 37 weeks gestations, aged < 28 days (*n* = 37)	• Positive relationships increase in EE and different levels of nursing interventions • Significant relationship between increased EE and decreased oxygen saturation
Salavitabar (2010) [33]	• Preterm infants: 29–33 weeks’ GA, admitted to NICU within 6 h of birth (*n* = 11)	• Mean HR during pre-care was directly associated with higher serum increases to sound stimuli • Serum cortisol during high sounds levels was significantly higher than during baseline sound levels • Changes in serum cortisol induced by sound associated with lower attention and behavioral responses
Peng (2011) [34]	• Preterm infants: < 37 weeks GA, aged < 28 days (*n* = 37)	• Positive correlation between stressors (noise, light, handling) and behavioral/physiological stress response • Correlation between behavioral stress indictors and physiologic stress signals (HR, RR, O_2_ saturation)
Smith (2011) [7]	• Preterm infants: < 30 weeks (*n* = 44)	• Average daily exposure to stressors was greatest in the first 14 days of life • Stressful exposures related to total brain injury score • Increased exposure to stressors associated with decreased brain size (frontal and parietal regions), altered brain microstructure, and functional temporal lobe connectivity • Alterations in neurobehavior at term equivalent associated with increased early stress
Liaw (2012) [35]	• Preterm infants (*n* = 30)	• Infant stress behaviors increased with more stress-inducing caregiving • Behaviors elevated in the absence of caregiving, indicating that the NICU environment is stressful • Infant regulatory behaviors occurred less frequently during intrusive caregiving • Lateral/prone position improved stability; non-nutritive sucking/prone positioning reduced disorganized behaviors
Slater (2012) [36]	• Preterm infants: < 37 weeks GA (n = 80)	• Tape removal associated with higher procedural pain scores • Tape removal associated with increase in oxidative stress • Oxidative stress associated with increased pain scores & decreased oxygen saturation
Cabral (2013) [37]	• Newborns main diagnosis of prematurity (n = 12), healthy newborns (n = 43)	• Salivary cortisol level in NICU infants elevated compared to healthy infants at home • Salivary cortisol decreased day 2—9, though comparison with the control group suggested persistent stress
Karpe (2013) [38]	• Intubated infants: 32—39 weeks GA (n = 32)	• Increased skin conduction noted for mechanical ventilation, ETT suctioning, fingertip punctures • Mean values during aspiration and finger-tip puncture differed considerably from those during mechanical ventilation • Values noted during aspiration comparable to fingertip puncture
Lin (2014) [39]	• Preterm infants (n = 37)	• Positive correlation between different levels of energy expenditure and seven stress behavioral responses • Positive correlation between different levels of nursing interventions and stress behaviors
Peng (2014) [40]	• Preterm infants: < 37 weeks GA, aged < 28 days (n = 37)	• Significantly positive relationship between EE and increased level of nursing intervention • Significantly negative relationship between EE and oxygen saturation
Provenzi (2016) [41]	• Very preterm infants < 32 weeks GA (n = 37), healthy term controls (n = 53)	• Blunted stress reactivity at 3 months in premature infants compared to controls • Number of skin-breaking procedures predicted stress-related reactivity, adjusting for confounders • Infants exposed to fewer skin-breaking procedures with salivary cortisol response similar controls
Rohan (2016) [42]	• Infants admitted to the NICU at < 35 weeks (n = 59)	• 3,354 pain-associated procedures across 59 subjects • Average of 56.8 of procedures by 37-week PMA • Significant negative correlation between the number of skin-breaking procedures and 17-OHP values • No association was found between 17-OHP and total hours of assisted ventilation
Zeiner (2016) [43]	• Preterm neonates: 25–35 weeks PMA, with mild severity of illness (n = 30)	• Significant increases for both biological and behavioral stress responses from before to during the care
Cong (2017) [44]	• Preterm infants: 28 0/7 to 32 6/7 weeks GA (n = 50)	• Daily mean of 22.97 ± 2.30 procedures, 42.59 ± 15.02 h of chronic events in initial 4 weeks admission • Stress during initial 4 weeks significantly associated abnormal NNNS at 37 weeks PNA • Daily mean duration of parental contact (minutes) correlated with improved habituation scores
D'Agata (2017) [45]	• Preterm infants (n = 41)	• Infants experienced average of 46 stressful experiences per day • Significant correlations between total average stress experience and neurobehavioral outcomes • Associations between stress exposure, FKBP5 genotype, and neurobehavioral outcomes, suggest environmental factors act synergistically with genetic susceptibility to affect neurobehavioral outcome
Fumagalli (2018) [8]	• Preterm infants: < 32 weeks GA and/or < 1500 g birth weight (n = 56);	• NICU-related stress and DNA methylation from birth to discharge was positive and significant • DNA methylation associated with brain volumes of the anterior temporal lobe
Fusch (2018) [46]	• Preterm infants (n = 33)	• Increased oxidative stress in formula fed infants than in infants fed formula plus breastmilk or breastmilk only
D'Agata (2019) [47]	• Preterm infants: birth weight < 1500 g (n = 82)	• Infants experienced an average count of 1804 stress events (range 824–2306 events) • Approximately 43 stressful events per day • Positive correlation in stress scores and cortisol response in infants born ≥ 28 weeks of gestation, negative correlation in infants born < 28 weeks gestation
Maciel (2019) [48]	• Infants admitted NICU within 3 h of life	• Total of 9,948 painful/stressful procedures were recorded, average of 11.25 ± 6.3 per day per neonate • 11,722 pain-management and relief interventions were performed, 98% nonpharmacological strategies, 2% pharmacological • Each neonate received 235 pain-management and treatment interventions during hospitalization on average: 13 nonpharmacological interventions per day, 1 pharmacological intervention every 2 days
Bembich (2020) [49]	• Preterm infants (n = 21)	• Sequence of weighing and bathing effected on stress response, higher stress when weighing done first • Higher stress response during both weighing and bathing independently associated with a lower PMA
Lavanga (2020) [50]	• Preterm infants: < 34 weeks, and/or birth weight of < 1500 g	• Dysmature EEG was found in patients under stress • Enhanced cortical connectivity and increased brain–heart communication was correlated with a higher stress load, autonomic activity not associated to stress exposure • Model demonstrated background stress state and chronic exposure persisted in absence of intrusive methods or pain
Nist (2020) [51]	• Preterm infants: 28–31 weeks PMA (n = 71)	• Median of 24 skin-breaking procedures and 30 invasive procedures during the first 14 days of life • Stress exposure was not correlated with early neurobehavior
Zhao (2022) [52]	• Preterm infants: 28–32 weeks GA	• Total mean frequency of acute stress/pain 614.12 ± 214.84, daily mean frequency 24.99 ± 7.13, daily mean of chronic events 41.13 ± 17.81 h during initial 28 days of admission • Higher NISS scores associated with higher NNNS stress, lower self-regulation and quality of movement
Van Dokkum (2022) [53]	• Preterm infants: < 30 weeks and/or birth weight < 1,000 g (*n* = 45)	• Mean daily NISS score was 66.5 (SD 8.7), with highest scores in the first 7 days of life • Slowest decrease in scores for lowest GA, particularly for nursing scores, compared to skin-breaking, monitoring and imaging, or medical morbidity-related score
Brekke (2023) [54]	• Preterm infants: 23–41 weeks GA (*n* = 72)	• Salivary cortisol correlated negatively with hours of parental presence (r = − 0.40, p =.01). In the linear regression, GA (B = −0.03, p =.02) and central venous lines (B = 0.23, p =.04) contributed to the variance in salivary cortisol in addition to parental presence
Li (2023) [55]	• Mechanically ventilated neonates in NICU (*n* = 80)	• Increased salivary cortisol with high-risk (endotracheal suctioning, arterial blood sampling), moderate-risk (venipuncture, gastric tube insertion), and low-risk procedures (bathing, diaper change) • Significantly larger fluctuations of salivary cortisol in high-risk procedures compared to low risk • Top influences on infant HR were arterial venipuncture, intubation, ETT suctioning, GT insertion
Nist (2023) [56]	• Preterm infants: 28- to 31-weeks GA (*n* = 60)	• Average 98 (SD = 41.8) invasive procedures in first two weeks of life • Nasal or oral suctioning (58.1%) followed by skin-breaking procedures (32.6%) were most frequent • Number of invasive procedures varied by maternal race; fewer total procedures in infants born to Black mothers • Significant variation in the number of invasive procedures among the four NICUs included
Van Dokkum (2023) [57]	• Preterm infants (*n* = 45)	• Evaluated rates of methylation of 7 stress-related and neurodevelopmentally genes • Rate of methylation for OPRM1 statistically different between day 7–14 and discharge
Xing (2023) [58]	• Preterm infant: 28–34 weeks GA (130)	• Acute stress exposure predicted the neurodevelopmental abnormalities in communication function • Chronic stress exposure associated with the problem-solving function
Bruce (2024) [59]	• Term newborns (*n* = 202) and their mothers	• Greater neurobehavioral stress signs at birth correlated with fewer social-communicative gestures at 18 months of age
Silva (2024) [60]	• Preterm neonates: < 37 weeks GA admitted to open bay (OB) or single-family (SFU) NICU designs	• Mean number of stressful procedures similar in both unit designs OB: 66 ± 35; 14—159, SFR 63 ± 29; 27—137 • Infants in the SFR design exhibited less stress behaviors and more self-regulation behaviors than those in the OB
Pavlyshyn (2024) [61]	• Preterm infants: ≤ 34 weeks (*n* = 56) GA	• Communication scores at 24–30 months independently negatively correlated with cortisol level, NICU LOS, and hospital LOS • Increase in cortisol above 0.64 mcg/dl predictive of personal social delay • Infants who suffered neonatal sepsis had increased risk of communication and gross motor delay
Van Dokkum (2024) [62]	• Preterm infants: < 30 weeks and/or birth weight < 1,000 g (*n* = 45)	• Higher stress exposure correlated with more breathing and mood-related problems in the second half-year of life

*Abbreviations*:* DNA* Deoxyribonucleic acid, *ETT* Endotracheal tube, *GA* Gestational age, *GT* Gastric tube, *HR* Heart rate, *HRV* Heart rate variability, *LOS* Length or stay, *PCA* Post conceptual age, *PICU* Pediatric intensive care unit, *PMA* Postmenstrual age, *NICU* Neonatal intensive care unit, *NISS* Neonatal Infant Stress Score [63], *NNNS* NICU Network Neurobehavioral Scale, *VLBW* Very low birth weight, *17-OHP* 17-hydroxyprogesterone

^*^Included procedures: endotracheal suctioning, heelstick, venous angio-catheter insertion, diaper change, positioning and bottle-feeding

^**^Nursing interventions were classified into five levels: level 1: interventions that include noise or light stimulation; level 2: interventions that include both noise and light stimulation; level 3: interventions that include noise or light and handling stimulation; level 4: interventions that include noise, light, and handling stimulation; and level 5: any intervention that causes pain

**Table 2:** Infant Stressors by category as identified in empiric literature

Stressor Category		Author (Year)
**Environmental**	Generalized Environment	Zahr (1995) [14]
		Yamada (2007) [28]
		Peng 2009 [32]
		Cabral (2013) [37]
		Peng (2014) [40]
		D'Agata (2017) [45]
		Cong (2017) [44]
		D'Agata (2019) [47]
		Lavanga (2020) [50]
		Nist (2020) [51]
		van Dokkum (2022) [53]
		Zhao (2022) [52]
		van Dokkum (2023) [57]
		Xing (2023) [58]
		Silva (2024) [60]
		van Dokkum (2024) [62]
		Bruce (2024) [59]
		Pavlyshyn (2024) [61]
	Light	Peng (2009) [32]
		Peng (2011) [34]
		Peng (2014) [40]
	Noise	Trapanotto (2004) [21]
		Salavitabar (2010) [33]
	Parental Separation	Cong (2017) [44]
		Brekke (2023) [54]
**Pain and Noxious Stimuli**	Skin breaking procedures (e.g., heel stick, peripheral venous catheter insertion)	Grunau (2005) [22]
		Ahn (2006) [23]
		Holsti (2007) [26]
		Pineles (2007) [27]
		Carbajal (2008) [29]
		Lucas-Thompson (2008) [30]
		Padhye (2009) [31]
		Karpe (2013) [38]
		Lin (2014) [39]
		Provenzi (2016) [41]
		Cong (2017) [44]
		Fumagalli (2018) [8]
		Maciel (2019) [48]
		Nist (2020) [51]
		Lavanga (2020) [50]
		Van Dokkum (2022) [53]
		Li (2023) [55]
		Nist (2023)[56]
	Mechanical Ventilation	Quinn (1998) [18]
		Matsubasa (2002) [20]
		Yamada (2007) [28]
	Endotracheal Tube Suctioning	Bernert (1997) [15]
		Ahn (2006) [23]
		Karpe (2013) [38]
		Li (2023)[55]
	Other Noxious Stimuli	Carbajal (2008) [29]
		Slater (2012) [36]
		Peng (2014) [40]
		Rohan (2016) [42]
		Li (2023) [55]
		Nist (2023) [56]
**Routine Care**	Routine Handling (e.g. positioning, physical assessment)	Zahr (1995) [14]
		Holsti (2007) [26]
		Peng (2009) [32]
		Smith (2011) [7]
		Peng (2011) [34]
		Liaw (2012) [35]
		Lin (2014) [39]
		Peng (2014) [40]
		Zeiner (2016) [43]
		van Dokkum (2022) [53]
	Bathing	Peters (1998) [17]
		Lee (2002) [19]
		Liaw (2006) [24]
		Bembich (2020) [49]
		Li (2023) [55]
	Weighing	Neu (1997) [16]
		Bembich (2020) [49]
	Diaper Changes	Mörelius (2006) [25]
		Lin (2014) [39]
		Li (2023) [55]
	Feeding	Ahn (2006) [23]
		Fusch (2018) [46]

### Sample population and evaluation of infant stress

Nearly universally studies focused on preterm infants admitted to neonatal intensive care units. Of the 51 studies reviewed, 47 were conducted in neonatal intensive care units, 45 of which exclusively focused on premature infants. Three studies included infants cared for outside neonatal intensive care units, one focused on infants admitted to the pediatric intensive care environment. Studies focusing on near-term or term infants hospitalized in intensive care environments or on infants cared for in general pediatric intensive care or cardiac intensive care settings were lacking (Table 1). Studies typically excluded infants with genetic or congenital anomalies. No studies were identified that included infants with congenital heart disease.

Table 3 presents measures of infant stress utilized across reviewed studies. The measures can be classified as vital sign assessment (e.g., heart rate, respiratory rate), biochemical markers (e.g., serum or salivary cortisol, urine 8-Hydroxydeoxyguanosine, epigenetic methylation), biophysical assessment (e.g., skin conductance, energy expenditure, heart rate variation), observational scales (e.g., Neonatal Infant Stress Scale (NISS), Anderson Behavioral State Scale, Premature Infant Pain Scale), or clinical outcomes (e.g., NICU length of stay, number of skin breaking procedures). Observational scales were most utilized to evaluate infant stress (29 studies). Vital sign alterations were used in 13 studies, biochemical markers of infant stress were used in 14 studies, and 9 studies utilized biophysical evaluation. Most commonly, studies utilized a single measurement type to evaluate infant stress or stress response (32 studies), while two concurrent measurement types were utilized in 14 studies and three types of stress measurement concurrently utilized in 2 studies. The remaining three studies evaluated the epidemiology of painful procedures in hospitalized infants.

**Table 3:** Types of measurement for evaluation of infant stress utilized in empiric literature

Category & Specific Metric	Author (Year)
**Biochemical**	
Serum cortisol	Grunau (2005) [22]; Holsti (2007) [26]; Pavlyshyn (2024) [61]
Salivary cortisol	Mörelius (2006) [25]; Cabral (2013) [37]; Provenzi (2016) [41]; Brekke (2023) [54]; Li (2023) [55]; Bruce (2024) [59]
Hair cortisol	Yamada (2007) [28]
Serum Uric acid	Slater (2012) [36]
Malondialdehyde	Slater (2012) [36]
DNA methylation	Fumagalli (2018) [8]
Urine melatonin	Bruce (2024) [59]; Pavlyshyn (2024) [61]
Urine 8-oxodG/creatinine	Fusch (2018) [46]; Matsubasa (2002) [20]
Serum catecholamine (adrenaline & noradrenaline)	Quinn (1998) [18]
17-hydroxyprogesterone	Rohan (2016) [42]
**Biophysical**	
Heart rate variability	Grunau (2005) [22]; Padhye (2009) [31]
Energy expenditure	Lin (2014) [39]; Peng (2009) [32]; Peng (2014) [40]
Skin conductance	Zeiner (2016) [43]; Karpe (2013) [38]; Salavitabar (2010) [33]
EEG	Lavanga (2020) [50]
EMG muscle tension	Trapanotto (2004) [21]
**Vital Signs**	
Heart rate	Lin (2014) [39]; Bernert (1997) [15]; Lee (2002) [19]; Lucas-Thompson (2008) [30]; Peng (2011) [34]; Peters (1998) [17]; Zahr (1995) [14]; Peng (2014) [40]; Zeiner (2016) [43]; Pineles (2007) [27]
Respiratory rate	Peng (2011) [34]; Zahr (1995) [14]; Zeiner (2016) [43]
Oxygen saturation	Bernert (1997) [15]; Lee (2002) [19]; Peng (2011) [34]; Peters (1998) [17]; Zahr (1995) [14]
NIRS	Bernert (1997) [15]
MAP	Bernert (1997) [15]
**Observational Scale**	
Neonatal Facial Coding System	Grunau (2005) [22]; Holsti (2007) [26]
NICU Network Neurobehavioral Scale	Cong (2017) [44]; Zhao (2022) [52]
Anderson Behavioral State Scale	Zahr (1995) [14]; Ahn (2006) [23]
Premature Infant Pain Profile	Slater (2012) [36]; Mörelius (2006) [25]
Neonatal Infant Pain Scale	Mörelius (2006) [25]
NIDCAP Assessment Tool	Zeiner (2016) [43]
Assessment of Preterm Infants’ Behavior	Pineles (2007) [27]
Prechfl’s Behavioral State	Bernert (1997) [15]
Scafidi State Scale	Lee (2002) [19]
Leuven Pain Scale	Lavanga (2020) [50]
Neonatal Infant Stress Scale	Cong (2017) [44], D'Agata (2017) [45]; D'Agata (2019) [47]; Smith (2011) [7]; van Dokkum (2022) [53]; Zhao (2022) [52]; van Dokkum (2023) [57]; Xing (2023) [58]; Silva (2024) [60]; van Dokkum (2024) [62]
Study specific scale	Lin (2014) [39]; Lucas-Thompson (2008) [30]; Peng (2011) [34] Peters (1998) [17]; Bembich (2020) [49]; Liaw (2006) [24]; Liaw (2012) [35]; Neu (1997) [16]
**Clinical Metrics**	
ICU length of stay	Fumagalli (2018) [8]
Number of skin breaking procedures	Fumagalli (2018) [8]; Carbajal (2008) [29]; Maciel (2019) [48]
Days of mechanical ventilation	Fumagalli (2018) [8]
Number of painful procedures	Carbajal (2008) [29]; Cong (2017) [44]; Maciel (2019) [48]; Nist (2023) [56]
Days of enteral feeding	Fusch (2018) [46]

*EEG* electroencephalogram, *EMG* electromyography, *ICU* intensive care unit, *MAP* mean arterial blood pressure, *NIDCAP* Newborn Individualized Developmental Care and Assessment Program, *NIRS* near-infrared spectroscopy, *N-PASS* Neonatal Pain, Agitation, and Sedation Scale

### Environmental stressors

For the purposes of this review, environmental stressors were defined as aspects of the intensive care environment that may trigger a physiological or behavioral stress response. Studies evaluating the epidemiology of stressful encounters for infants in the intensive care environment reported high frequencies of stressful encounters. Infants across studies experienced approximately 43 to 75 stressful experiences per day of hospitalization [29, 47, 53, 45]. Stressors were shown to be concentrated in the first week of admission and consist of frequent handling, light, and noise in addition to painful procedures [7, 53, 34]. Markers of stress, including hair and salivary cortisol concentrations, have been shown to be significantly increased in infants in intensive care environments compared with healthy infants [28, 37]. Serial sampling of salivary cortisol concentrations in infants cared for in intensive care settings showed evidence of persistent stress, as levels did not downtrend comparably to those of healthy infants [37, 50].

Studies specifically evaluating the impact of noise stimuli demonstrated positive correlations between noise and both behavioral and physiological metrics of stress in NICU infants [21, 33, 40]. Despite this evidence, noise levels in intensive care settings frequently exceed 45 decibels adjusted (dBA), the level recommended by the American Academy of Pediatrics for neonatal intensive care unit settings, and interventions (e.g. visual noise feedback system, incorporation of unit-wide “quiet times”) to limit noise levels do not reliably demonstrate noise reduction [64]. Noise is often associated with nursing interventions, increasing sensory stress on infants [14, 32, 40]. Handling for routine nursing interventions has been identified as an additional stressor for infants in intensive care settings, with infants in open warmers being at higher risk for frequent handling than those in incubators [14, 34, 39, 47]. Additional light exposure often occurs with nursing interventions [32, 34] and is hypothesized to be an additional environmental stressor. Light cycling interventions have been tested with variable results [65], though no studies were identified in this review specifically delineating the impact of light stimuli on markers of infant stress. While the maintenance of normothermia is particularly important in infants being cared for in intensive care settings [66–68], no studies reviewed evaluated the impact of temperature changes on biological or behavioral markers of infant stress. However, infant temperatures outside the 36.5 to 37.5 °C range recommended by the World Health Organization of cold stress have been shown to negatively impact infant outcomes, including leading to increased adverse events [67] and late-onset sepsis and mortality [66].

Factors related to unit design, visitation policies, and culture of care were also related to environmental infant stress. In a comparison of NICU unit design, open bay design was correlated with increased infant stress behaviors and poorer self-regulation that single-family-room designs despite similar exposure to stressful encounters in both groups indicating that environmental protection may promote biobehavioral regulation [60]. Two studies evaluated parent-infant interaction and infant stress. An evaluation of COVID-19 parental visitation restrictions, parental presence, and infant stress found infant salivary cortisol negatively to be correlated with parental presence [54]. Hours of parental presence appeared to mitigate the neurobehavioral impact of pain-related stress [44]

### Routine care

Specific direct-care nursing activities that evoke an infant stress response but do not require skin-breaking are discussed below. Across studies, both physiological and behavioral measurements of stress increased in response to noninvasive direct care nursing activities requiring infant handling, e.g. bathing, weighing, physical assessments, and diaper changes [16, 17, 19, 24–26, 35, 36, 39, 43, 49, 55]. Several studies specifically evaluated bathing, reporting a significant impact on physiological stability with changes in heart rate, oxygen saturation, and increased frequency of infant stress behaviors [17, 19, 24]. Obtaining an infant’s weight, which often accompanies bathing, is another nursing activity shown to be positively related to infant stress states [16, 49]. Two studies evaluated procedural differences of weighing in response to infant stress. Neu and Browne (1997) [16] found that weights obtained while infants were swaddled induced less stress, and Bembich et al. [49] reported lower stress response when bathing preceded weighing.

Across studies exploring the impact of direct care nursing activities on infant stress level, higher levels of caregiving e.g. nursing interventions that require handling or cause pain such as adhesive removal, have been associated with more severe stress response [24, 36, 39, 55], though even low-level nursing interventions, such as obtaining a weight or diaper changes, have been demonstrated as stressful [25, 55]. When compared to healthy infants, those in the neonatal intensive care unit demonstrated higher levels of salivary cortisol with routine diaper changes [25]. Infant positioning and composition of enteral feeding were also associated with infant stress signals, with infants cared for in a prone or hammock position demonstrating less frequent disorganized behaviors and more frequent regulatory behaviors [35], and higher levels of oxidative stress measured by urine 8-Oxo-2′-deoxyguanosine in formula-fed infants than in infants receiving breastmilk [46]. Despite being generally regarded as innocuous, these routine nursing interventions and care activities have demonstrated a consistent pattern of an induced stress response on both physiological and behavioral indices. The impact of environmental stressors on infant growth and development is likely multifactorial but may be related to the increased energy expenditure associated with environmental stress [40]. In addition to the positive correlation of environmental stressors with behavioral and physiological stress response, a significant positive relationship has been noted between energy expenditure and intensity level of nursing intervention [39, 40].

### Pain and noxious procedures

Direct care activities or interventions performed on infants requiring actual or potential tissue damage therefore likely to elicit pain responses, are discussed below. Twenty studies investigated exposure to painful or noxious stimuli in the context of behavioral or physiological indices of infant stress.

Exposure to painful procedures in hospitalized infants is high. Across studies evaluating the frequency of painful procedures, infants were subjected to an average of 11 to 24 painful procedures per day [42, 44, 48, 37]. Nist et al. (2020) reported a median of 24 skin-breaking procedures and 30 invasive procedures in the first 14 days of life in a cohort of 71 preterm infants 28 to 31 weeks postmenstrual age (PMA). Frequency of painful encounters was higher earlier in hospital admission [37, 42]. In a large observational multisite study, neonates (< 28 days of life) including term infants and preterm infants, admitted to intensive care settings underwent 98 painful interventions in the first 14 days of admission [29]. Repeat procedural attempts were common, particularly for central venous catheter and peripheral arterial catheter insertion which required 4 or more attempts in 18% of infants [29]. Similarly, Rohan (2016) found that premature infants admitted < 35 weeks gestation were exposed to an average of 57 pain-associated procedures by the 37th week of PMA [42]. The variation in painful encounters reported across studies is notable and indicating that the culture of care may impact infant stress exposure. This is supported by Nist et al. (2023) which reported significant variation in the number of invasive procedures across four NICUs after adjusting for birthweight and illness severity [56].

Procedures and interventions commonly cited as painful across studies included skin-breaking procedures, e.g. heel sticks, venipuncture, and endotracheal suctioning [8, 15, 22, 27, 30, 31, 37, 38, 42, 48, 55, 56]. Across studies, skin-breaking procedures were associated with more intense infant stress response measured by behavioral indices, physiological metrics including cardiovascular response, and neuroendocrine response including serum and salivary cortisol concentrations [22, 23, 27, 30, 31, 38, 41, 55]. Infants requiring mechanical ventilation had higher concentrations of urinary 8-hydroxydeoxyguanosine, a marker of oxidative stress [20], as well as sympathetic nervous system activation measured by skin conductance fluctuation [38]. The duration of mechanical ventilation and specific mode of ventilation have been identified as contributory to infant stress states [8, 20, 18, 28]. Similarly, endotracheal suctioning correlated with an increased infant stress response across studies [15, 23, 38, 55]. Evaluation of infant cortisol response during routine procedures in intubated preterm infants reported the largest fluctuation in cortisol to be associated with high-risk procedures (endotracheal suctioning, arterial blood sampling), though even low risk procedures (bathing and diaper changes) were associated with increased salivary cortisol compared to sleep state [55].

### Impact of infant stress exposure

Sources of stress infants encounter in the intensive care environment have been associated with acutely increased physiological and behavioral indices of stress and long-term neurodevelopment deficits. Increased exposure to stressors has been associated with epigenetic changes [8, 57], increased neurological injury [7], abnormal cerebral development [7], and neurobehavioral outcomes in preterm infants [44, 52]. Infants exposed to painful stressful stimuli experienced neuro-dysregulation i.e. dysmature electroencephalogram (EEG) patterns characterized by discontinuity, persistence of slow waves, and general lack of smoothness, that persisted in the absence of intrusive stimuli [50]. Exposure to painful stress stimuli impacted short-term stress response in infants [22], blunted cortisol reactivity at 3 months [41], and long-term epigenetic modification of genes associated with regulation of stress and neurodevelopment [8, 58]. Epigenetic modifications, such as methylation, are biochemical changes to DNA influencing gene expression without altering the nucleotide sequence. An exploratory study of methylation of stress-related and neurodevelopmentally relevant genes in preterm infants suggested cumulative stress exposure may positively correlate with epigenetic methylation at NICU discharge [57]. A positive correlation was found between neonatal intensive care unit-related stress exposure, increased epigenetic methylation of the serotonin transporter gene from birth to discharge, and infant brain volume after 43 weeks PMA in preterm infants [8].

Studies evaluating the longitudinal impact of infant stress suggest negative associations persist throughout the first years of life. Stressful encounters in the neonatal period have been associated with alternations in language development and communication [58, 59, 61], mood disorders [62], and respiratory problems [62]. In term infants, higher stress signs at birth were associated with fewer social-communicative gestures at 18 months of age [59]. In preterm infants < 34 weeks gestational age, communication scores at 24 to 30 months were independently negatively correlated with cortisol level, neonatal intensive care unit length of stay, and hospital length of stay [61]. Higher stress exposure in preterm infants has been correlated with increased risk of abnormalities in early communication behaviors e.g. vocalization, social smile and problem solving at 3 months [58] as well as more respiratory problems and mood problems reported at 12 months [62]. These findings suggest that stressors encountered in the clinical environment impact infants beyond the acute period of stress, likely into childhood and possibly adulthood.

## Discussion

### Summary of evidence

This scoping review identified stressors beyond disease processes encountered by infants in intensive care environments reported in empiric literature. Identified stressors fell into three major categories: environmental stressors, routine care, and pain or noxious stimuli. These numerous stressors are likely to occur concurrently or in proximity, compounding their impact, resulting in a substantial cumulative stress burden. Positive correlations between clinical interventions and infant stress response were found across studies and routine care, generally regarded as less invasive or even benign e.g. diaper changes, were associated with infant stress response. Current evidence suggests that the stress response triggered by these encounters early in life is toxic, negatively impacting both short-term physiological stability and long-term neurodevelopmental function.

This review is the first structured summary of recent literature focused on identification of specific stressors encountered by infants in intensive care environments, including those related to the intensive care environment, provision of care, and pain. In a prior review by van Dokkum and colleagues focused on the impact of stress in premature infants born less than 37 weeks, negative associations between infant stress incurred during neonatal intensive care admission and neurocognitive outcomes, epigenetic alterations, and endocrine functioning were reported [2] Most studies included in this review focused on pain-related stressors i.e. skin-breaking procedures; no studies were identified which met inclusion criteria that investigated environmental stressors such as noise or light exposure [2]. This review is the first structured summary of recent literature focused on identification of specific stressors encountered by infants in intensive care environments that includes those related to the intensive care environment, provision of care, and pain. Comprehensive identification and awareness of specific stressors encountered by infants cared for in intensive care environments provides a foundation to limit stress-related morbidity in infants cared for in neonatal, pediatric, or cardiac-focused intensive care units.

Studies evaluating of stressors encountered by infants cared for in non-neonatal ICU settings, i.e., pediatric or cardiac intensive care units, are lacking. Moreover, many studies identified in this review focused exclusively on otherwise health premature infants, specifically excluding infants with congenital anomalies, systemic infections, or those undergoing surgical procedures. No studies were identified focusing on specific stressors in the direct care environment encountered by infants with congenital heart disease (CHD).

Stress assessment variables varied across studies, underscoring the lack of a universal standard in assessment of infant stress. Behavioral observation scales and vital sign alterations were the most used metrics. Biochemical metrics, including DNA methylation; cortisol measurements from serum, saliva, or hair; and serum catecholamine concentrations were increasingly utilized in more recent studies. Skin conductance, an indirect measure of sympathetic autonomic activity, and physiological markers such as heart rate, heart rate variability, and arterial saturation were also commonly assessed. In many studies, a biological or physiological stress metric in conjunction with a behavioral assessment tool was utilized, though behavioral assessment tools also varied across studies. Studies aimed at standardization of stress assessment with a tool that thoroughly incorporates physiological, biochemical, and behavioral measures are warranted.

This structured review has some limitations. Only peer-reviewed articles published in or translated to English were eligible for inclusion. Additionally, the variation in stress assessment across studies does not allow for direct comparison or combination of study results. Finally, included studies were largely conducted in premature infants in neonatal intensive care settings, and consequently, results may be less generalizable to critically ill term or near-term infants cared for in other settings.

### Clinical implications

In high-stakes clinical settings, every action likely has a consequent and potentially detrimental reaction. Findings indicate that infants cared for in intensive care settings experience, in addition to pain and noxious stimuli, environmental and care-related stressors. Many activities which may be considered routine in the intensive care unit may trigger a stress response. These varied sources of stress, which are related to both nursing and medical interventions, create a substantial cumulative infant stress burden, exposure to which likely translate to lasting negative impact. Awareness and quantification of specific sources of infant stress may allow interprofessional teams to implement stress reduction strategies in the intensive care setting including quantification of stressful encounters and enhanced care coordination between medical and nursing teams. Interdisciplinary models of care aimed at limiting cumulative stress burden in critically ill infants may promote physiological stability and improved short- and long-term outcomes.

### Future research

Animal models suggest that maternal separation early in life impacts acute stress response and epigenetic regulation of longitudinal stress responses [69, 70]. Two studies were identified empirically evaluating parental separation as a source of stress in infants indicating that reduced infant-parent interaction may exacerbate infant stress during intensive care hospitalization. Future work focused on association of parental absence on stress markers for critically ill infants and the impact of parental presence in mitigating stress response when infants encounter stressors in the intensive care environment may guide models of care which limit maternal separation and optimize parental involvement in the care of critically ill infants.

There was a notable lack of studies focused on the evaluation of stressors in infants with CHD, presenting an area for future investigation. Though the studies reviewed here overwhelmingly focused on premature infants cared for in neonatal intensive care units, the stressors identified are common to the management of critically ill infants and likely to impact all infants cared for across intensive care settings. Across studies, the exposure to stressors varied widely. Future investigations focusing on the influences contributing to variability in infant stress exposure, including patient, clinician, and environmental factors, would inform the development of effective stress reduction models in intensive care.

Infants with CHD commonly encounter most of the stressors identified in this review and likely experience short- and long-term consequences as a result. However, the epidemiology of stressful encounters in infants with CHD undergoing surgical repair and the resultant impact remain unknown. Unlike preterm infants who typically experience high levels of stressful interventions in the first two weeks of admission that decreases over the course of hospitalization [7, 57], infants with CHD likely encounter a similar initial stress burden that is then punctuated by a high stress surgical procedure and subsequent recovery. Additionally, infants undergoing cardiac surgery almost universally require mechanical ventilation at some point in their hospitalizations and are often cared for outside of temperature-controlled isolettes, which may further increase their stress exposure in comparison to healthy preterm infants [8, 14, 18, 20, 28]. The unique and highly invasive clinical course required for surgical correction or palliation of CHD predispose these children to substantial environmental, direct care, and pain-inducing stress stimuli, which is in addition to the surgical stress associated with operative repair. The widespread presence of infants with CHD in contemporary neonatal, pediatric, and cardiac intensive care units offers research opportunities to define stress exposure in this patient population and determine its consequences. Further, identification of modifiable stressors could lead to initiatives aimed at reducing stress burden to optimize outcomes in this high-risk subset of critically ill infants.

## Conclusion

Review of current literature reveals that infants in intensive care environments face a multitude of stressors—including environmental factors, routine care-related stress, and painful procedures—that often overlap or occur in proximity, contributing to a significant cumulative stress burden. These stressful encounters have profound short- and long-term physiological and neurodevelopmental impacts. Studies to date overwhelmingly focus on preterm infants in neonatal intensive care settings, with limited research addressing the stressful experiences of infants with CHD, highlighting a crucial area for future investigation. Identification and mitigation of stressors encountered by infants in intensive care environments is fundamental to improving patient care and optimizing recovery in this vulnerable population.

## Data Availability

Not applicable.
